# Anæsthesia—Nitrous Oxide

**Published:** 1890-10

**Authors:** 


### Anæsthesia—Nitrous Oxide.

"An Analysis of a Series of One Thousand Nitrous Oxide Administrations Recorded Systematically." Paper by Dr. J. F. Silk, London, England.

He impressed the importance of recording systematically, for by such means alone could satisfactory explanations of curious anomalies in practice be hoped for. To appreciate and explain action of drug upon human body it is essential to compare three methods of investigation, viz.; physiological, pathological, and clinical.

Post-mortem records limit the pathological records. Physiological investigation with clinical observations was the desideratum.

In majority of cases mental and physical conditions prior to administering anæsthetics, the circumstances were trivial. In twenty-one neurotic troubles were marked—this does not include simple nervousness. Two cases—a family history of insanity—both cases troublesome during administration; three cases subject to epileptic fits—one described epileptic aura commenced; four cases phthisis, nothing out of ordinary; one case valvular disease of heart; lividity following longer than usual, and a tendency to syncope.

Diabetes, one case, no change in urine. Pregnancy, nine cases, nothing gone wrong, but a tendency to vomit; one case during lactation; billious attack next day; sixty-five records of consecutive administrations—*i. e.*, where patient was allowed to recover consciousness and then, after a few minutes, submitted to the anæsthetic; in twelve per cent. more or less retching; in two instances asphyxial symptoms; nine per cent. marked hysteria. In six out of nine cases of remote effects the records were unfavorable, but in seventy-three per cent. nothing to call for remark as to remote or immediate effects. Average quantity of gas used between four and five gallons; average time per case to anæsthetize sixty-seven and five seconds; duration of anæsthesia variable; absence of conjunctival reflex or presence of jactitations no guide. Method of using gas—44 cases the gasometer, 467 gas administered pure, 502 occasions a supplemental bag was employed. Believes the use of supplemental bag tended to increase onset of anæsthesia.

Phenomena: Circulatory; increase of pulse; loss of tidal wave; accentuation of dicrotic curve; increase of heart force.

Muscular system: Rythmic movements of arms and legs.

Opisthotonos most common in females. Eyes pupils, average size before 5.5. mm.

In 366 cases decided dilatation during anæsthesia, but this is not to be relied upon as a test for narcosis.

Micturition occurred in ten cases; all females; erotic movements and sexual illusions in six cases; five unmarried females, and one early in pregnancy.

Great trouble in getting records of after effects, but probably more or less headache was the rule rather than the exception.

